# Supplementary material for: OLR1 Is a Pan-Cancer Prognostic and Immunotherapeutic Predictor Associated with EMT and Cuproptosis in HNSCC
Source: Int J Mol Sci. 2023 Aug 17;24(16):12904. doi: 10.3390/ijms241612904 (PMC10454104; doi:10.3390/ijms241612904)
Supplement: Supplementary file 1 [file ijms-24-12904-s001.zip › Figure Caption.pdf]

Figure S1: Forest plot showing the correlations between *OLR1* expression and PFI (A) and DSS (B) in various cancers in the TCGA database.
Figure S2: Correlation between *OLR1* expression and immune cell infiltration in various pan-cancer types using (A) XCELL, (B) MCPCOUNTER, and (C) QUANTISEQ algorithms.
Figure S3: Heatmap showing correlations between *OLR1* expression and RNA levels of 39 EMT-related genes in pan-cancer using Spearman's correlation test.
Figure S4: Protein–protein interaction network for *OLR1* using the STRING dataset.
