# Supplementary figures and images for: OLR1 Is a Pan-Cancer Prognostic and Immunotherapeutic Predictor Associated with EMT and Cuproptosis in HNSCC
Source: Int J Mol Sci. 2023 Aug 17;24(16):12904. doi: 10.3390/ijms241612904 (PMC10454104; doi:10.3390/ijms241612904)

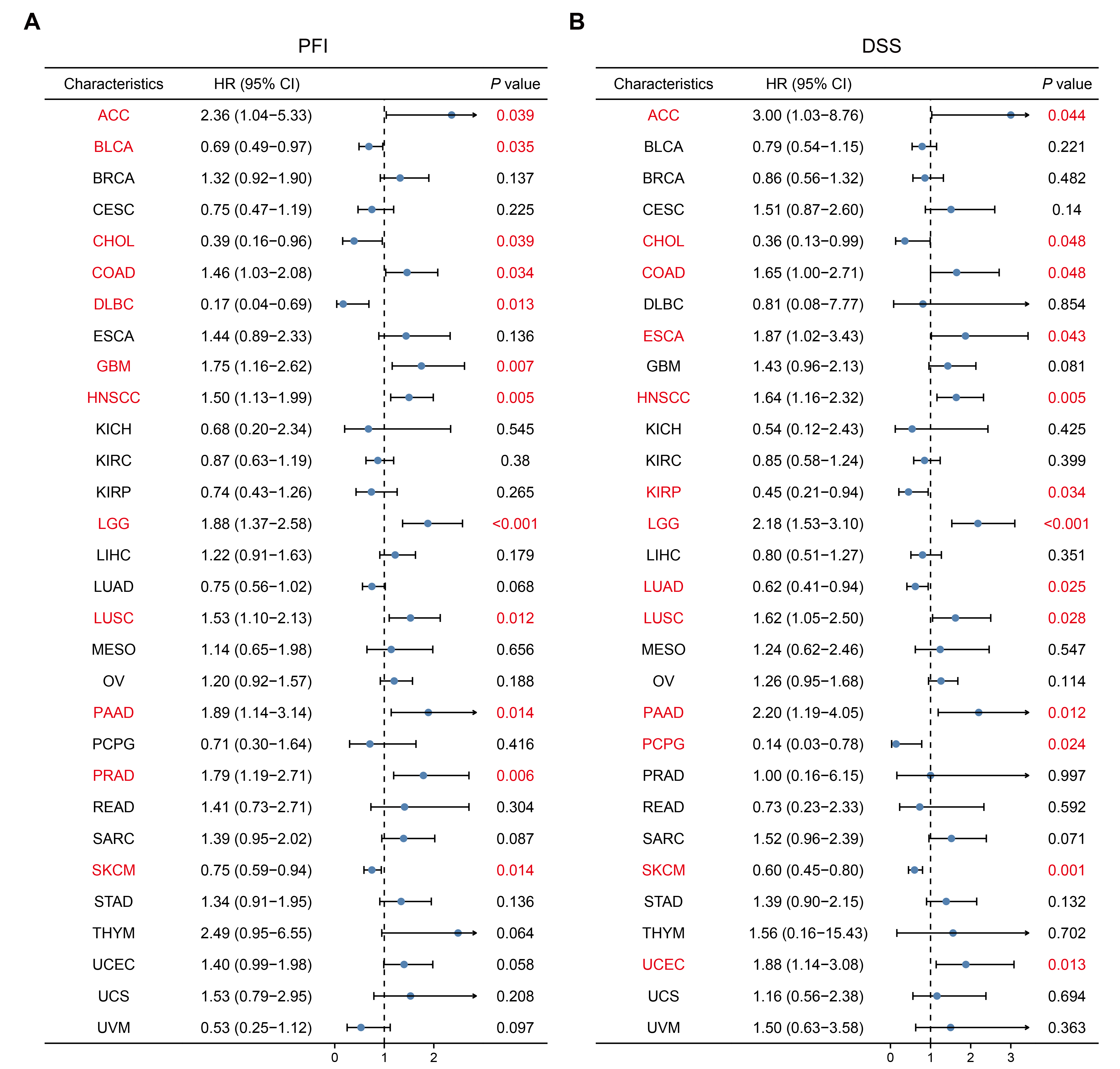

Supplement: Supplementary file 1 [file ijms-24-12904-s001.zip › Supplementary_FigureS1.tif]

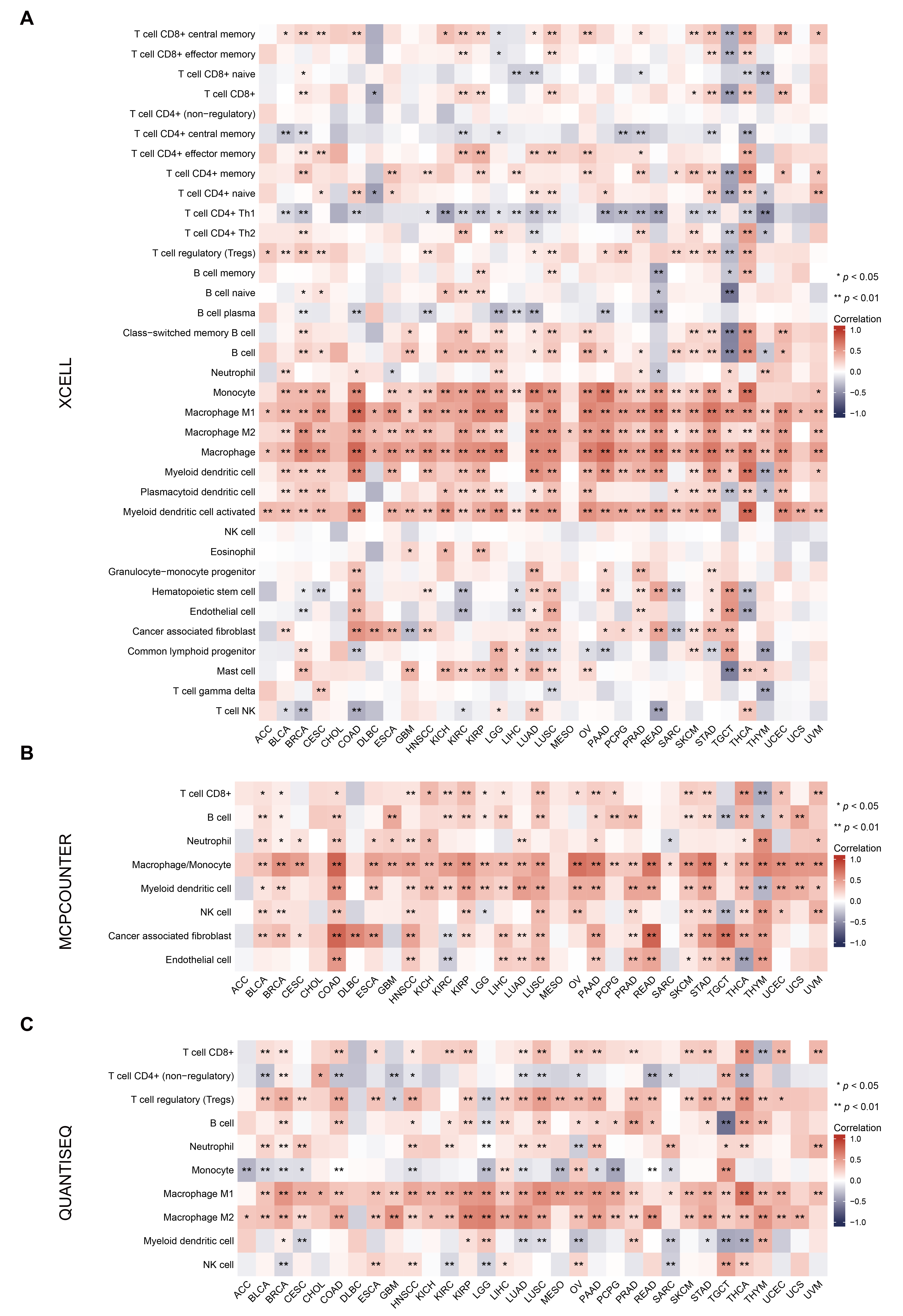

Supplement: Supplementary file 1 [file ijms-24-12904-s001.zip › Supplementary_FigureS2.tif]

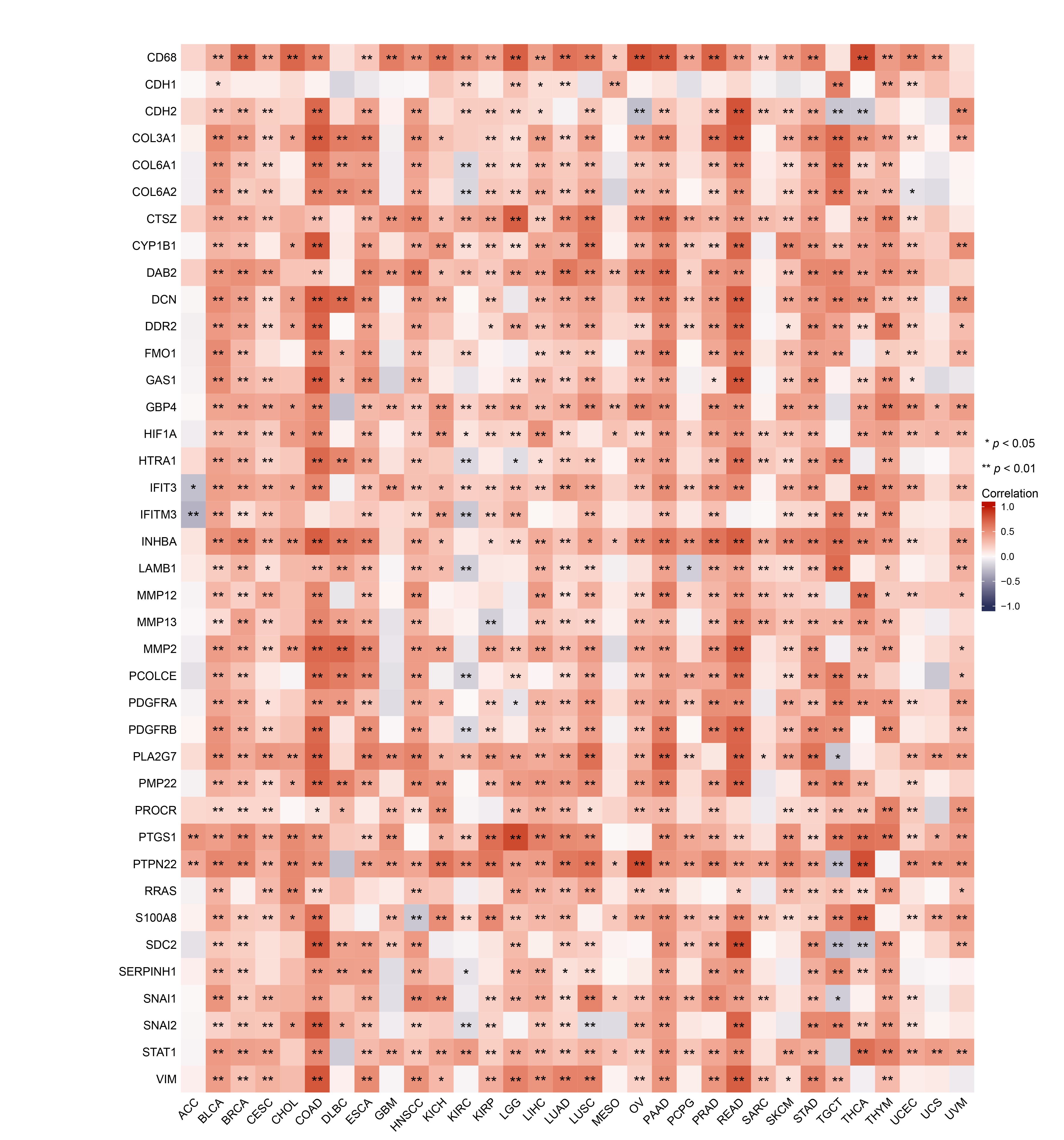

Supplement: Supplementary file 1 [file ijms-24-12904-s001.zip › Supplementary_FigureS3.tif]
